# Characterization of coronary vein motion in patients with low and moderate ejection fractions

**DOI:** 10.1186/1532-429X-13-S1-P232

**Published:** 2011-02-02

**Authors:** Jonathan Suever, Pierre Watson, Stamatios Lerakis, Robert Eisner, Robert O'Donnell, John Oshinski

**Affiliations:** 1 Georgia Institute of Technology/Emory University, Atlanta, GA, USA; 2 Emory University School of Medicine, Atlanta, GA, USA

## Objective

To quantify periods of low motion of the coronary veins in patients with low and moderate EF in order to optimize acquisition of whole-heart coronary magnetic resonance venograms (cMRV).

## Background

Three-dimensional, whole-heart, navigator-gated, contrast-enhanced MRI techniques are used to acquire cMRVs which can be used for planning lead placement in cardiac resynchronization therapy (CRT). In order to reduce cardiac motion artifacts, it is desirable to set the trigger delay time to acquire image data only during periods of low vessel motion, typically assumed to be during diastole. By knowing the temporal length and location of the low motion period, the acquisition window can be optimized for coronary vein imaging. Previous studies have examined coronary *artery* motion, but an analysis of the coronary *veins* has not been performed.

## Methods

32 total patients were analyzed. These patients were considered in 3 groups: 13 patients scheduled for CRT (LVEF<35%, QRS>120ms), 6 patients with low LVEF but normal ECG (LVEF<35%, QRS<120ms) and 13 patients with moderate LVEF and normal ECG (LVEF>35%, QRS<120ms).

Steady-state free procession (SSFP) cine, two-chamber, long-axis images were acquired with at least 30 frames over the cardiac cycle using a 1.5T Siemens Avanto or Philips Intera.

The centroid and cross-sectional area of the coronary sinus were determined for each phase. Low motion periods were characterized by frame-to-frame displacements less than 0.5 mm. Based on the ratio of systolic to diastolic low motion period durations, patients were classified as either having a *systolic* or *diastolic* rest period.

## Results

The coronary sinus was visible in the two-chamber cine images in all patients. 38% of the patients had *systolic* rest period and 62% had a *diastolic* rest period.

**Figure 1:**
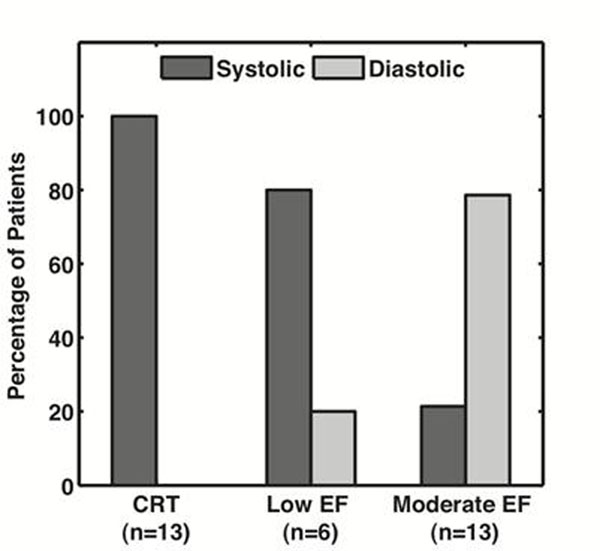
Percentage of patients having longer systolic or diastolic rest periods.

The major findings of this study were: 1) 95% of patients with LVEF<35% had a longer *systolic* rest period (including 100% of CRT candidates) Figure 1, 2) 85% of patients with moderate EF (>35%) had a longer *diastolic* rest period, 3) 4 patients with a longer *systolic* rest period had no diastolic rest period.

## Conclusion

A longer rest period and therefore a preferable imaging window occurs during systole for patients with low LVEF regardless of QRS duration. Diastole is a preferred window for patients with moderate LVEF. Each patient’s low motion periods should be categorized before imaging the coronary veins to ensure the correct period is being utilized in regard to low motion duration.

